# Supplementary material for: Tyrosine Kinase Inhibitors Are Promising Therapeutic Tools for Cats with HER2-Positive Mammary Carcinoma
Source: Pharmaceutics. 2021 Mar 6;13(3):346. doi: 10.3390/pharmaceutics13030346 (PMC8002158; doi:10.3390/pharmaceutics13030346)
Supplement: Supplementary file 1 [file pharmaceutics-13-00346-s001.pdf]

pharmaceutics

Supplementary Materials: Tyrosine Kinase Inhibitors Are Promising Therapeutic Tools for Cats with HER2-Positive Mammary Carcinoma

Andreia Gameiro, Filipe Almeida, Catarina Nascimento, Jorge Correia and Fernando Ferreira

Table S1. Immunohistochemic characterization of the FMC cell lines (CAT-M, FMCp and FMCm), and the human breast cancer cell line (SkBR-3).

Cell Line	ER *	PR **	Ck5/6	Ki-67 (%)
**CAT-M**	3	8	NEGATIVE	50.2
**FMCp**	7	0		57.4
**FMCm**	3	0		68.5
**SkBR-3**	0	4		53.6

* ER—Estrogen receptor; ** PR—Progesterone receptor.

Table S2. Analysis of the *her2* TK domain mutations in the feline mammary carcinoma clinical samples. DNA samples were acquired from tumor mammary tissues, and PCR and sequencing steps were performed with specific primers.

Nucleotide Position	Mutations Frequency	Breed [1] (n)	Age Class [2] (n)	Size Class [3] (n)	Tumor Subtype (n)	Stage (n)	mRNA (bp); aa Modification	Observations *
19572 C > A	3	Ind	2 (2); 3	2 (3); 3	LB (2); TN	3 (2); 4	2153; T > K	Substitution (Polar neutral to polar basic)
19573 A > C	4	Ind (3); SM	2	2; 3 (3)	LB (3); TN	2; 3 (3)	2154; T	Synonymous
19575 G > T	1	Ind	2	2	LB	2	2156; E > D	Substitution (Polar neutral to polar acid)
19581 G > T	2	Ind	2	2; 3	LB	2; 3	2162; R > M	Substitution (Polar basic to apolar)
19584 A > G	3	SM	2 (2); 3	2 (2); 3	TN; LB-HER2; LB	2; 3 (2)	2165; K > E	Deletion AAG to -AG
19584 / 19585 AG > CC	1	Ind	2	2	LB	2	2165 / 2166; K > T	Substitution (Polar basic to polar neutral)
19587 T > G	1	Ind	2	2	LB	2	2168; V > G	Substitution (both apolar)
19593 T > C	1	Ind	2	2	LB	2	2174; V > A	Substitution (both apolar)
19604 G > T	1	SM	2	3	LB	3	2185; G > STOP	Substitution
19607 G > A	1	Ind	2	3	LB	3	2188; A > T	Substitution (Apolar to polar neutral)
19626 Del	1	Ind	2	3	TN	3	2207; K > R	Deletion AAG to A-G
19631 A > T	1	Ind	2	2	LB	2	--	Intron
19643 A > C	2	Ind (2)	2 (2)	2;3	LB (2)	2; 3	--	Intron
19666 A > T	1	Ind	2	2	LB	2	--	Intron
19667 G > T	1	Ind	2	2	LB	2	--	Intron – Homozygous sample Increased mRNA levels (n= 1)[#]
19676 G > A	3	Ind (3); SM	2 (3)	2 (2); 3	LB (2); TN	2; 3; 4	--	Intron
19677 T > C	3	Ind (3)	2 (2); 3	2 (3)	HER2; LB (2)	2 (2); 3	--	Intron
19712 A > C	2	Ind (2)	2 (2)	2; 3	LB (2)	2; 3	--	Intron
19713 A > T	2	Ind (2)	2 (2)	2; 3	LB (2)	2; 3	--	Intron

19714 G > A	4	Ind; SM	2 (2)	2; 3	LB-HER2; LB	3 (2)	--	Intron
19716 G > C	1	Ind	2	3	LB	3	--	Intron
19822 G > A	1	NF	2	2	LB	3	--	Intron
19828 G > C	1	Ind	2	2	LB	2	--	Intron
19845 G > T	1	Ind	2	2	LB	2	--	Intron
19858 G > T	1	Ind	2	2	LB	2	--	Intron
20044 G > A	2	Ind (2)	1; 2	3; 1	HER2; LB-HER2	3; 1	--	Intron
20045 A > G	2	Ind	2	3	HER2	3	--	Intron
20142 G > A	17	Ind	1	1	LB-HER2	1	--	Intron Increased mRNA levels (n=2) #
20278 T > C	5	Ind (5)	2 (2); 3 (3)	1; 2 (3); 3	LB (5)	1; 3 (3); 4	--	Intron
20289 G > A	12	Ind (9); SM (2); Per	2 (5); 3 (7)	1 (2); 2 (8); 3 (2)	LB (5); LB-HER2 (2); TN (3); HER2 (2)	1 (3); 2 (4); 3 (5)	--	Intron
20380 C > G	5	SM (3); Per; Ind	2 (3); 3 (2)	1; 2 (3); 3	LB (2); LB-HER2; TN (2)	1; 2; 3 (3)	2404; P > A	Substitution (both polar neutral)
20382 T > C	1	Per	3	1	TN	1	2406; P	Homozygous; Synonymous
20384 A > T	1	Per	3	1	TN	1	2408; Y > F	Substitution (Polar neutral to apolar)
20385 T > G	2	Ind	3; 2	1; 2	LB; LB-HER2	1; 2	2409; Y > STOP	Substitution
20428 G > C	2	Ind	1; 3	1 (2)	LB-HER2	1	2452; G > R	Substitution (Apolar to polar basic)
20436 G > A	1	Ind	1	1	LB-HER2	1	2460; E	Synonymous
20459 A > T	1	Ind	1	1	LB-HER2	1	2483; E > L	Substitution (Polar neutral to apolar)
20531 C > T	4	Ind; SM; Per (2)	2 (3); 3	1; 2; 3 (2)	TN (2); LB (2)	1; 3 (2); 4	--	Intron Increased mRNA levels (n=1) #
20533 G > A	2	Ind (2)	2; 3	1; 3	HER2; LB-HER2	1; 3	--	Intron
20612 G > A	1	Ind	2	3	HER2	3	--	Intron
20827 A > G	7	Ind (7)	2 (3); 3 (4)	1; 2 (4); 3 (2)	LB (7)	1; 3 (5); 4	--	Intron Equal mRNA levels (n=3) #; Increased mRNA levels (n=4) #; Decreased mRNA levels (n= 1) #
20940 T > G	34	Ind (28); SM (4); Per (2); NF	1 (2); 2 (18); 3 (14)	1 (8); 2 (17); 3 (9)	LB (17); LB-HER2 (8); TN (7); HER2 (2)	1 (8); 2 (5); 3 (18); 4 (3)	2716; W > G	Substitution (both apolar)

[1] Ind—Indeterminate; SM—Siamese; NF—Norwegian Forest Cat; [2] Age class 1< 8 years; 2—8 to 12 years; 3> 12 years; [3] Size class 1 < 2 cm; 2—2 to 3 cm; 3 > 3 cm; * All the mutations not specified were heterozygous; # Reported by Ferreira et al. 2019 [32].

Table S3. Feline cell lines presented a few number of mutations in the feline *her2* TK domain, and none of them were described in women, as leading to TKi resistance. DNA samples were acquired from the cells in culture, and PCR and sequencing steps were performed with specific primers.

	CAT-M			
	**18/19**	**19/20**	**20/21**	**21/22**
**Intron (bp)**	19677	20142	--	20827
	T > C	G > A		A > G

			FMCp				
	**18/19**	**19/20**	**20/21**	**21/22**		**20 ***	**21**
**Intron (bp)**	19677	20063	20560	20804	**Exon (bp)**	20312	20657 G > C
	T > C	T > G	A > G	A > G		G > A	(Arg to Gly)
		20077	20584	20816		20327	20684 C > T
		G > T	T > C	T > C		C > A	(Arg to Cys)
		20091		20822		20339	20716 C > G

		A > G		C > A	A > G	(Leu to Val)
		20105		20831	20348	
		G > A		C > T	T > C	
		20136		20857	20354	
		C > G		C > A	T > C	
		20141			20366	
		C > T			T > C	
		20175			20405	
		A > C			T > C	
		20183			20408	
		G > A			T > C	
		20218			20450	
		G > T			A > G	
		20242			20436	
		G > C			C > T	
		20257				
		T > C				
		**FMCm**				
	**18/19**	**19/20**	**20/21**	**21/22**		
**Intron (bp)**	19677<br>T > C	20142<br>G > A	--	20827<br>A > G		

* Synonymous mutations.
